# Research on Reverse Osmosis (RO)/Nanofiltration (NF) Membranes Based on Thin Film Composite (TFC) Structures: Mechanism, Recent Progress and Application

**DOI:** 10.3390/membranes14090190

**Published:** 2024-09-05

**Authors:** Huibin Geng, Weihao Zhang, Xiaoxu Zhao, Wei Shao, Haitao Wang

**Affiliations:** 1 School of Material Science and Engineering, Tiangong University, Tianjin 300387, China; 2 School of Chemical Engineering and Technology, Tiangong University, Tianjin 300387, China; 3 School of Environmental Science and Engineering, Tiangong University, Tianjin 300387, China; 4 State Key Laboratory of Separation Membranes and Membrane Processes, Tianjin 300387, China

**Keywords:** thin film composites, membranes, interfacial polymerization, modification methods

## Abstract

The global shortage of clean water is a major problem, even in water-rich regions. To solve this problem, low-cost and energy-efficient water treatment methods are needed. Membrane separation technology (MST), as a separation method with low energy consumption, low cost, and good separation effect, has been widely used to deal with seawater desalination, resource recovery, industrial wastewater treatment, and other fields. With the continuous progress of scientific and technological innovation and the increasing demand for use, NF/RO membranes based on the TFC structure are constantly being upgraded. This paper presents the recent research progress of NF and RO membranes based on TFC structures and their applications in different fields, especially the formation mechanism and regulation of selective layer structures and the modification methods of selective layers. Our summary provides fundamental insights into the understanding of NF and RO membrane processes and hopefully triggers further thinking on the development of membrane filtration process optimization.

## 1. Introduction

The growing scarcity of freshwater resources has seriously affected the normal life of one-third of the world’s population, and there is an urgent need to find ways to purify water available for use from non-conventional sources to address this problem [1]. The development and advancement of alternative water production technologies is a promising solution to the water scarcity dilemma. Membrane separation technology, which separates, classifies, purifies, or enriches two or more components, is recognized as a technology with the potential to strategically change industrial technology [2]. Thin film composite (TFC) polyamide (PA) membranes are pressure-driven separation membranes for the separation of low molecular weight substances or polyvalent and monovalent ions [3]. Starting from 1978, when Cadotte et al. prepared the first thin film composite nanofiltration membrane on ultrafiltration (UF) membrane by interfacial polymerization (IP) for high-efficiency brackish water desalination, it has been widely researched and developed due to the high efficiency of separation and low economic cost. Currently, the fields of seawater desalination, industrial wastewater treatment, resource recycling, and drinking water purification are essential processes and have become the most popular separation membrane process in the field of separation [4,5,6].

**Figure 1:**
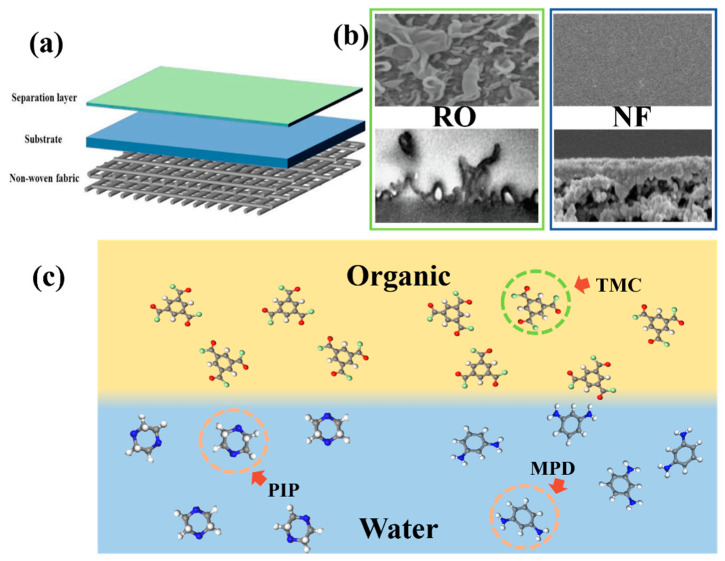
(**a**) Three-layer structure of TFC membrane [13]; (**b**) Selective layer morphology of NF and RO membranes [14,15]; (**c**) Schematic diagram of the interfacial polymerization process.

NF/RO membranes based on the TFC structure have a relatively dense structure on the surface, with certain similarities in the preparation method and material selection [7]. Both NF/RO membranes are composed of a non-woven layer, a support layer, and a selective layer, and their structures are shown in Figure 1a. The non-woven layer serves to provide high mechanical strength to the membranes. The support layer is mainly made of polysulfone, polyethersulfone, polyacrylonitrile, cellulose acetate, and other polymers prepared by the phase transformation method, which plays a role in providing mechanical strength and porous support substrate [8,9,10]. The topmost selective layer, which plays the main separation role, is usually polyamide and polyester and is generally prepared by interfacial polymerization. Although the change of each layer will cause a change in membrane properties, the regulation of the selective layer is relatively simple compared with the other two layers, so it is more effective to modify and precisely control the selective layer of TFC membranes in order to achieve the optimal performance of the membranes. However, since the rapid and intense IP reaction causes great difficulties in studying the formation mechanism of selective layers, the study and modification of polyamide separator layers are of great significance in improving the performance of membranes [11,12].

Although TFC-structured NF/RO membranes have become the most promising separation technology for water purification and treatment, the preparation process and application technology have become very mature. However, the research and development of membranes have always attracted the attention of scientists and research scholars as the traditional synthesis methods and materials used still make membranes face challenges such as susceptibility to fouling, difficulty in improving performance, and low service life [16,17]. This review summarizes the synthesis mechanism of PA selective layers, the modulation mode, the research progress of membranes, their applications in different fields, and the challenges faced. It provides insights into the further understanding of TFC-structured NF/RO membranes and triggers further thoughts on the development and research of membrane separation technology.

## 2. Mechanism of PA Layer Formation

**Figure 2:**
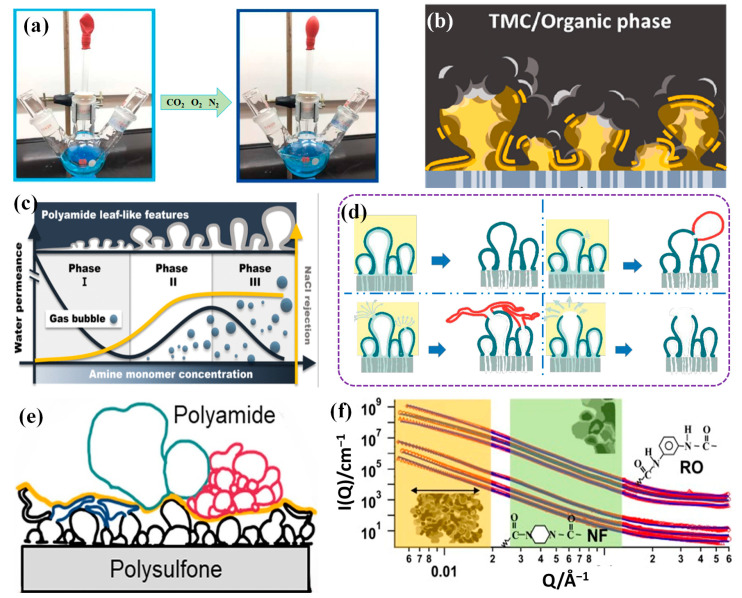
(**a**) Escape of dissolved gases during the IP reaction; (**b**) Effect of solvent vaporization on the structure; (**c**) Effect of gases on the membrane structure; (**d**) Effect of the number of reacting monomers on the PA structure; (**e**) Structures of the five PA layers; (**f**) Small-angle diffraction technique used to analyze the generation of the PA structure.

PA selective layers based on TFC structures are prepared from amine monomers and acyl chloride monomers through IP reaction, as shown in Figure 1b. For NF membranes, the PA selective layer is relatively smooth, whereas the selective layer of reverse osmosis membranes has a very pronounced “ridge-and-valley” structure, as shown in Figure 1b. Due to the high reactivity of monomers in RO membranes, the IP reaction is violent, resulting in a ridge and valley structure being generated in the PA layer. So far, there have been many different accounts of the formation of this characteristic “ridge-and-valley” structure [18]. The most popular theories currently attribute the formation of the ridge and valley structure to the dissolved gases O_2_, CO_2_, and N_2_ dissolved in the aqueous phase solution, as shown in Figure 2a,c. During IP, these dissolved gases escape from the aqueous phase due to the exothermic nature of the reaction. The intense IP reaction produces a polyamide barrier that inhibits gas escape and encapsulates the gas molecules, which, therefore, occupy a certain amount of free space and form a ridge and valley structure [19]. Based on the molecular theory of dissolved gases, Ma et al. added NaHCO_3_ to the aqueous phase and used pH adjustment to decompose NaHCO_3_ to produce CO_2_ at different pH and obtained a distinct “ridge-and-valley” structure [20]. However, another theory suggests that it is not the gases dissolved in the aqueous phase solvent but the gases produced by the exothermic vaporization of the low-boiling solvent during the reaction that occupy the space leading to the formation of the “ridge-and-valley” structure. Moreover, when more gas is produced by vaporization, it may break through the ridge and valley structure that has been created and cause defects, as shown in Figure 2b [21]. In addition to the release of gases generated in the two-phase solvent, the pore size of the support membranes also affects the escape of gases [22]. When the pore size of the basement membrane is too large, the dissolved or to-be-released CO_2_ gas will escape from the bottom of the basement membrane, and the PA layer generated without additional material occupying the space will be relatively flat, without the appearance of the typical “ridge-and-valley” structure. When the support membrane aperture is small, the gas is not able to escape from the support membrane aperture, thus creating a “ridge and valley” structure.

Dissolved gas theory is not the only research theory that explains the “ridge-and-valley” structure formation. Song et al. suggested that the formation of a “ridge-and-valley” structure is mainly due to the amount of reactive monomers [23]. However, when the content of reactive monomers is only enough to satisfy the degree of polymerization with acyl chloride monomers, the resulting morphology will be relatively flat or form a small nodular structure. When the diamine monomer is in excess, during the reaction process, the amine monomer will diffuse too much into the oil phase and react with more acyl chloride to form more polyamide chains, which are stacked to form a “ridge-and-valley” structure, as shown in Figure 2d. When the diamine monomer is further increased, subsequent diamine monomers break through the already formed polyamide barrier, causing the already generated chain structure to break, thus further allowing the PA layer to continue to grow and generate a larger blade structure. Controlling the morphology of the PA selective layer by the amount of reactive monomers is the simplest way to regulate it, but it is also necessary to consider whether the changes in other properties of the membrane will affect the use of the membrane [24].

The formation of PA layer structures is explained from a microscopic point of view as a reaction that occurs at the molecular level, and understanding the generation of PA layer structures at the molecular level helps to make the formation mechanism clear [25]. Grzebyk et al. classified the formation of PA layers into different hierarchies and visualized the hierarchies as shown in Figure 2e, which describes in depth the formation mechanism at each step [26]. However, understanding the formation mechanism requires practical means of characterization to aid in illustration and reflect the veracity of the conclusions. Singh et al. used advanced small-angle diffraction to characterize the changes in the PA layer structure during its formation, explaining the growth of the PA layer structure from molecular stacking and interchain compaction to primary unit clusters [27]. Due to the rapidity of polymer growth in the primary phase of the IP reaction, monitoring the kinetics of the IP reaction can help to gain a deeper understanding of the generation of the PA layer structure, as shown in Figure 2f. Mansouri et al. used in situ fluorescence spectroscopy to explain the effect of reactive monomer concentration on the generation of PA layer structures, further refining the theoretical understanding of the generation of “ridge-and-valley” structure [28]. The use of a micro-controlled flow platform also provides a simpler and more intuitive way to study the kinetics of monitoring IP reactions. Nowbahar et al. monitored the progress of the IP reaction using a micro-controlled flow platform, which provided a clear understanding of the spatial distribution and reaction paths of the two reacting monomers, providing an intuitive means of understanding the IP process [29].

Due to the rapidity of the IP reaction, current monitoring platforms and tools do not allow for a thorough monitoring of the process. However, certain conclusions can be corroborated and inferred to a certain extent, providing new ideas and methods for understanding the process of IP. The high accuracy of optical technology is considered to be an emerging means of investigating IP reactions, and optical in situ characterization techniques are gradually being applied to investigate IP processes. For example, slow scattering and Rutherford scattering spectroscopy enable accurate data to be obtained on the effect of monomer concentration and IP time on the reaction process. In situ fluorescence and in situ UV techniques can be used to monitor the IP reaction from its inception, to study the rate of diffusion of monomers in the reaction, etc. Advanced characterization techniques allow the generation of PA layers to be studied from a deeper perspective, making it possible to precisely control the IP reaction. However, due to the technical limitations in applying it well to the study of IP reactions, advanced techniques still need to be combined with conventional characterization methods to be illustrative [30].

Although there are many different views on how the ridge and valley structure of the PA layer is formed, a great deal of research and innovation has been conducted based on current research theories on how to obtain membranes with in-demand properties. Although it is not possible to draw definitive conclusions about the generation mechanism of the PA layer structure with great clarity, the use of existing research has led to a continuous improvement and clarification of the reasons for the formation of the PA layer structure. With the deepening of the research and the development of the technology, the formation mechanism of the PA layer “ridge-and-valley” structure will surely be clarified gradually and be able to guide the preparation and development of membranes with excellent performance.

## 3. Modification Methods and Latest Research Progress

The ideal membrane meets the requirements and criteria of high flux, high retention, high mechanical strength, good acid and alkali resistance, chlorine resistance, and long-term stability [31]. However, the current NF and RO membranes with TFC structure still face one or more of the above problems and are difficult to break through. However, the TFC-structured NF and RO membranes prepared by IP have the advantages of being easy to prepare and having a low cost, and the performance of the membranes can be easily improved by selecting one of the angles for modification [32]. Membrane performance is usually assessed by water flux and retention rate, and all modification studies seek to improve both flux and retention rate. However, if the improvement of one index is pursued while the other index is lowered and cannot meet the use standard, it means that the performance of the membrane is running, and the membrane loses its application value. Therefore, for the modification of membrane performance, the improvement of two evaluation indexes should be taken into account at the same time. Understanding the mechanism of TFC structure formation is the most effective way to modify the membrane to enhance a specific property. The modification methods generally used from the initial selection of materials to the final IP reaction include the selection of novel reactive monomers, the selection of suitable additives and solvents, and the development of novel preparation methods. 

### 3.1. Application of New Monomers

Monomers have a decisive influence on the performance of TFC membranes; different monomer reactivity will lead to the formation of different morphologies of the membrane surface, so the performance of the membranes varies [33]. Membranes prepared from PIP with TMC are usually considered NF membranes, and membranes prepared from MPD with TMC become RO membranes. The functional groups and geometrical structure of the reactive monomers play a decisive role in the formation of PA layers concerning properties such as degree of crosslinking, hydrophilicity, charge, and pore size. Designing ideal reactive monomers at the molecular level helps to obtain membranes with ideal performance and functionality and helps to expand the application areas of membranes, fundamentally solving the limitations of membrane performance that are difficult to break through. The performance of membranes synthesized from conventional reactive monomers has been constrained by the interplay of water permeability (A) and water–salt permselectivity (A/B), also known as the trade-off effect [34]. The study of changes in reaction dosage and experimental conditions based on the original has been fully investigated, and it is difficult to make further improvements in the performance of the membrane. The development of new materials and the synthesis of new monomers are important directions and prospects for membrane breakthroughs in existing research [35]. This paper summarizes the new monomers that have been used in recent years and compares them with the traditional monomers, which are listed in Table 1. Although the use of new monomers has good prospects for the enhancement of membrane properties, further research should be carried out on the long-term stability and anti-pollution properties after the preparation of membranes for practical applications to achieve the purpose of industrialization or commercialization as soon as possible [36].

The preparation of membranes by translating the intrinsic properties of monomers into corresponding membranes is an important method [48]. Conventional monomers can be molecularly designed to be reactive monomers that change membrane properties, and monomers with free volume can be effective in increasing water transport channels through membranes. Suitable ratios can lead to an increase in the free volume between the polymers, interconnecting most of the voids between the resulting PA polymers, leading to a significant increase in the water permeation performance of the membrane [49]. Typical new reactive monomers are rigid substances that are inspired by gas separation membranes. Rigid monomers can increase the free volume between selective layers to enhance the permeation properties of the corresponding membranes and may also be involved in the reaction to influence the IP reaction to achieve membrane modification [50]. Villalobos et al. prepared NF membranes with high-performance TFC structures by reacting cyclodextrins with free volume as aqueous phase monomers with acyl chloride monomers. The use of the free-volume cavity size of cyclodextrins allows for the precise separation of specific substances [51]. In addition to the free volume of the monomer itself, its participation in the reaction process can also be used to influence the overall IP reaction. The use of substances with free volume together with conventional reactive monomers as reactive monomers is a more common modification method. Jiang et al. reacted two bisphenols with rigid twisted backbones together with PIP as aqueous monomers in TMC, which increased the free volume of the PA layer and the connected voids without causing defects due to the control of the special structure at the molecular level [52]. In addition to reactive aqueous phase monomers, organic-phase acyl chloride monomers with free volume can also be used. Tripterenes are used for the synthesis of highly permeable rigid monomers with bridged structures. Ali et al. modified a monomer with this structure as a bridged bicyclic trimethylenetetrayl tetrayl chloride as an oil-phase monomer for reaction with MPD, which enhanced the membrane flux to 9.2 LMHbar^−1^ compared with the original membranes [53]. Free-volume reactive monomers can effectively enhance membrane performance and form more stable micropores with intrinsic structure while enhancing membrane stability [54]. However, most of these substances are expensive and require relatively severe conditions of use, which not only increases the cost of preparation but also complicates the synthesis conditions [55]. To generalize the use of substances with free volume in the preparation of TFC-structured membranes, molecular modification of existing substances to give themselves free volume for use in enhancing membrane permeation performance should be considered. Also, reducing the price of the synthesis process for improved structural distortion of non-coplanar monomers is a challenge that needs to be addressed urgently.

### 3.2. Modification of Two-Phase Solution

The nature of the reaction solvent is also one of the most important factors affecting the membrane properties, as both the miscibility of the solvents and the nature of the solvent itself can influence the process and development of the IP reaction [56]. Since the solubility of MPD in the organic phase is much greater than that of TMC in the aqueous phase, the IP reaction occurs on the organic phase side of the miscible zone [57]. Therefore, changing the reaction solvent can increase or decrease the solubility of the MPD in the organic phase, thereby modifying the IP reaction process to improve the membrane properties. Usually, the solvents used for organic phases are aliphatic solvents such as hexane, heptane, etc. They have high volatility and very low polarity [58]. Difficulty in dissolving relatively complex substances and large differences in solubility coefficients with aqueous solvents limit the diffusion of amine monomers. Higher polarity organic solvents can improve the solubility of amine monomers in the organic phase and increase the miscibility in the miscible zone. Ma et al. used isomeric alkanes as solvents for the organic phase to improve the polarity and viscosity of the oil phase [59]. The viscous organic solvents not only provide a thin reaction interface while inhibiting the diffusion of the chlorinated monomers, but the obtained membranes have relatively high pure water flux and salt rejection. Park et al. replaced an aromatic solvent with an aliphatic solvent as the organic phase solvent. When toluene and xylene were used together as organic phase solvents, the size of the miscible zone was significantly increased, enabling the generation of a loose PA layer in addition to a dense PA layer, resulting in a salt rejection rate of 99.9% in the membrane [60]. The use of reaction solvents needs to consider the impact on the porous support layer; organic solvents will damage the surface of the porous support layer. The surface pores of the porous support layer that is not resistant to solvents will be altered, affecting the growth of the PA layer, and at the same time, its affordability should be considered in practical applications. 

**Figure 3:**
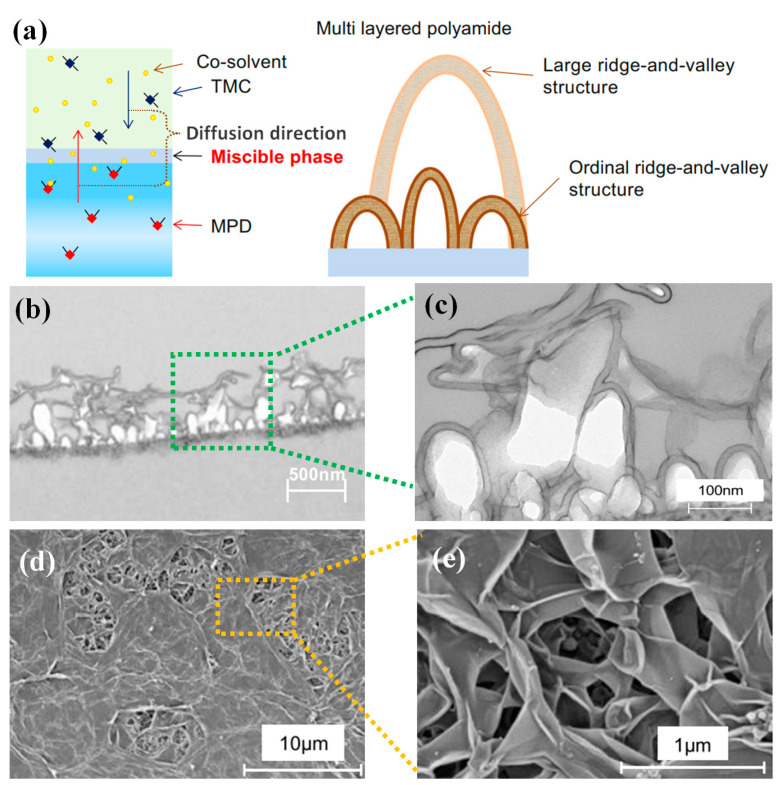
(**a**) Co-solvent-formed ridge and valley structure with roof-like shape; (**b**) low magnification SEM of membrane cross-section; (**c**) high magnification SEM of membrane cross-section: (**d**) low magnification SEM of membrane surface: (**e**) high magnification SEM of membrane surface [67].

The addition of reactive additives to the solvent can also change the nature of the IP reaction and effectively enhance the membrane properties. Commonly used additives include nanoparticles, co-solvents, and additional reactive substances. The most effective and commonly used method for controlling the solubility and diffusivity of amine monomers in the reaction zone is the introduction of a co-solvent into the organic or aqueous phase [61]. The influence of co-solvents as additives to the IP reaction is self-evident. The addition of co-solvents not only influences the nature of the solvents in the two phases and shortens the difference in solubility parameters between two solutions but also has a decisive effect on the distribution and solubility of the reacting monomers in the miscible zone and between the two phases [62,63]. Co-solvents can be divided into aqueous phase co-solvents and organic phase co-solvents. Due to the high affinity of the amine monomer for the organic phase and the limited affinity of the chloride monomer for the aqueous phase, the change in the organic phase affects the IP reaction more than the change in the aqueous phase and has a greater influence on the formation of the PA layer [64]. In addition, compared with the high addition rate of aqueous co-solvents (5–40%), organic-phase co-solvents need to be added at a rate of less than 5%, which makes them more suitable for membrane modification from the point of view of economic cost [65]. Khorshidi et al. introduced hydrophilic alcohols as co-solvents into the aqueous phase, generating thicker selective layers compared with the aqueous phase without added co-solvents [66]. Thanks to the similar solubility coefficient between the added alcohol co-solvent and the already formed PA polymer, it is possible to make the PA layer relatively more susceptible to swelling at the film surface. Organic phase co-solvents have higher compatibility with the aqueous phase and, therefore, add a miscible phase between the incompatible aqueous-organic phases, as shown in Figure 3. Within this miscible phase, the amine monomer reacts with the acyl chloride monomer to form a larger ridge and valley structure. When the miscible zone disappears after the co-solvent diffuses into the aqueous phase, the amine monomer and the acyl chloride monomer near the interface continue to react to form smaller ordered “ridge and valley” structures. Eventually, a so-called roof-like “ridge-and-valley” structure will be generated.

By varying the amount and type of co-solvent, it is possible to control the morphology and free volume of the resulting selective layer, thus controlling the membrane properties. However, excess co-solvent destabilizes the interface and enlarges the thickness of the reaction zone. At the initial stage of the reaction, the expansion of the reaction zone may cause the dispersed PA oligomers to be farther apart from each other, and the initial PA layer may also be slightly swollen by the co-solvent in the reaction zone, both of which together result in the formation of a relatively loosely separated layer. However, the addition of aqueous-phase co-solvents and organic-phase co-solvents can significantly improve the IP reaction conditions and change the morphology of the selective layer and the film properties. However, the difference in dosage and functionality between the two is still unexplored. In the future, the differences between the two-phase co-solvents need to be clarified to realize the possibility of synergistic interactions between the co-solvents working together to improve membrane properties.

In addition to affecting the nature of the IP reaction process, the inherent nature of the additives themselves will also affect the performance of the membrane. For example, when nanoparticles are used as additives, they not only affect the IP process but also end up being present within the resulting PA layer to increase the permeability properties of the membrane [68]. Nanoparticles that are often used as fillers include inorganic particles such as silver oxide, carbon nanotubes, and MOFs. Al-Hobaib et al. introduced silver oxide nanoparticles of different sizes into the preparation of RO membranes, which were embedded in the PA layer through the reaction of two-phase monomers. The addition of silver oxide makes the surface of the membranes smoother and improves the hydrophilicity significantly, which increases the water flux of the membrane accordingly [69]. Ji et al. used UIO-66 with different potentials as an additive in the preparation of PA layers, and the reticular nanostructure formed by UIO-66 was utilized to increase the pure water flux of the membrane (0.6 MPa 58.5 LMH) [70]. Although nanomaterials have a considerable effect on the enhancement of membrane performance, most of the nanoparticles have poor compatibility with the PA matrix, which tends to cause non-selective voids and ruin the membrane’s performance. To improve the compatibility of nanoparticles with the matrix, modification of nanoparticles can help their application in enhancing membrane properties. Grafting the surface of nanoparticles and modifying groups is another advantage of applying nanoparticles for membrane performance enhancement. This is because these materials are able to increase the pure water flux through the membrane by adding water channels. The so-called water channels are solid-state channels in which the material itself has fast water transport properties while providing a restricted space for selective transport of small molecules at the sub-nanometer scale [71]. The molecular sieving effect induced by nano water channels plays an important role in the whole membrane separation mechanism. Because of its own cavity size, it can provide a fast channel for the passage of water molecules to improve the water permeability, while the hydrate formed by ionic solutes is retained to ensure stable salt rejection [72]. Shao et al. modified a positively charged GO surface grafted with two amines into aminated nanoparticles, and the modified GO showed no phase incompatibility with the PA polymer. The permeation performance of the membrane is enhanced by the nature of graphene oxide’s channels, and the surface-modified amino group also enhances the membrane’s resistance to fouling [73]. The addition of nanoparticles has gained a bright future for the enhancement of membrane performance, but careful consideration should be given to the recycling and degradation of membranes after they have been disposed of and whether there is any damage to the environment [74]. 

Additional reactive substances are added to the original amine and acyl chloride reactive monomers to participate in the reaction together. The addition of additional reactive substances will participate in the IP reaction, resulting in the presence of two reactions in the vicinity of the miscible zone, which, due to the difference in reaction rates and exothermicity, will result in more zones with a thermal gradient. This enhances the instability of the interface, and the two reactions produce structurally different polymers that also produce more regions of excessively large molecular potential gradients that enhance the instability of the interface, favoring the generation of surface voids after the selection of the layer. Liu et al. introduced 1-methylimidazole as an aqueous phase additive into the aqueous phase, which can react with TMC to form imidazole salts [75]. This reaction increases the instability of the IP reaction and favors the formation of a PA layer with a pore structure at the back, leading to a thinner and denser selective layer. However, when both reactions coexist, the complexity of the reaction increases, making it difficult to control the IP reaction accurately.

### 3.3. New Modification Methods

The enhancement of membrane properties using conventional preparation methods is limited to changing variables such as the type of monomer concentration, reaction time, and reaction solvent. Novel preparation methods can overcome the limitations of the original film-making methods. These include the addition of intermediate layers, reverse IP, electrostatic spraying, unsupported IP, and simplified preparation routes [76,77]. TFC-structured membranes are prepared by IP between aqueous and organic phase solutions on a porous support material. The porous substrate determines the amount of aqueous-phase monomers that can participate in the IP reaction, as well as the structural morphology formed [78]. To avoid limiting the amount of reactive amine monomers due to the hydrophobicity of the porous substrate, a suitable intermediate layer is usually inserted between the substrate and the selection layer. The intermediate layer not only improves the storage and distribution of amine monomers in the aqueous phase but also controls the diffusion of reactive monomers and serves as a template for the IP reaction to achieve customized membrane surface structures [79]. More rigid nanoparticles are usually chosen as intermediate layers, such as MOFs and carbon nanotubes. Wen et al. introduced two-dimensional MOF as an intermediate layer on the surface of polyethersulfone membranes, which fully compensated the geometrical defects of the support membranes. The diffusion of the diamine monomer during the reaction is accelerated, which increases the crosslinking degree of the membranes but significantly reduces the thickness of the PA layer, increasing the flux by a factor of two while maintaining the salt retention rate [80]. Rigid substances such as MOF have a considerable effect on the enhancement of membrane properties. However, there is still the possibility of incompatibility with PA, which can cause non-selective voids, leading to a loss in membrane performance. Synthetic composite interlayers can avoid this defect while also acting as growth templates to control the generation of selective layers. Chen et al. coated flexible polymer gelatin enriched with hydrophilic groups coated on the surface of a base film, which provides growth sites for rigid nanoparticle zirconium phosphate. The composite intermediate layer formed by gelatin and zirconium phosphate resulted in a more homogeneous distribution of the amine-reactive monomers, which were inhibited from further growth by the formation of a dense PA layer, resulting in the growth of a thinner selective layer [81].

The presence of an interlayer not only influences the IP reaction but can also be used to its advantage to play a positive role in the transport of water through the membrane. The choice of a hydrophilic interlayer facilitates water transport by using its hydrophilic properties. Hydrophilic cellulose was used as an intermediate layer by filtration by Wang et al. Enables increased storage of diamine monomers and slows down IP, resulting in lower crosslinking. At the same time, due to the high hydrophilicity of the interlayer, which has a “drag effect” on water transport, the synergistic effect of the two gives the membrane a considerable pure water flux of up to 204 L·m^−2^·h^−1^ [82]. The synergistic improvement of membrane properties using both the intrinsic properties of the intermediate layer and the influence of the IP reaction process can provide a new route to the preparation of ideal separation membranes.

The reverse IP reaction involves reversing the order of addition of the aqueous phase solution and the organic phase solution by pouring the organic phase solution onto the surface of the support membranes and then pouring the aqueous phase solution. The reversed-phase IP reaction can effectively avoid the risk of hydrolysis of the chloride monomer in the organic phase. Song et al. used reverse IP to form structures with crater morphology on the membrane surface. Compared with the “ridge-and-valley” structure formed by conventional IP, this type of morphology does not form a large blade structure. However, it can improve the separation efficiency and significantly reduce the surface roughness of the membrane to improve the fouling resistance of the membranes [83]. The reverse IP reaction requires that the water in the pores of the polymer support membranes is first replaced with an organic solvent using the appropriate solvent to prevent hydrolysis of the acyl chloride monomer. This process requires the complete replacement of water in the pores of the support membranes. It is often necessary to fully saturate the support membranes with the participating organic phase solvent after the solvent has been replaced.

The unsupported IP reaction uses an aqueous phase as a base and then pours in an organic phase solution. After the formation of a polyamide membrane within a certain period, the two-phase solution is removed, and the already-formed PA layer is placed on the surface of the substrate [84]. Unsupported IP does not require consideration of the effect of the substrate on the distribution and storage of amine monomers. This is equivalent to reducing the experimental conditions for conventional IP reactions and making it easier to achieve controlled IP reactions. The relatively free aqueous solution leads to unrestricted diffusion and distribution of the amine monomer. The ability to dissipate heat faster during the interfacial reaction increases the kinetic rate of the reaction, which in turn generates a smoother selective layer [85]. Traditionally, the ability of the selective layer to bind to the support layer is achieved by interchain entanglement of the polyamide polymer generated between the amine monomer and the chloride monomer. However, the unsupported IP reaction does not involve the support layer, so the bonding strength between the selective layer and the support layer is much lower than that of the traditional IP. However, consideration can be given to applying external pressure to reinforce the bonding strength between the selection layer and the support layer. For example, a polyamide layer polymerized at a separate interface can be vacuum-filtered onto the surface of a substrate. Avoiding the influence of the substrate on the IP reaction while increasing the bond strength between the selective layer and the support layer [86].

Inspired by reverse IP and independent IP, the aqueous phase solution and the organic phase solution can be repeatedly alternately reacted. Layer-by-layer self-assembly of selective layers to achieve smoother or thicker film surfaces [87]. Gu et al. reacted with an aqueous solution of MPD and an n-hexane solution of TMC both repeatedly and alternately, and the self-assembly of the selective layer was accomplished with spin-coating [88]. The resulting PA layer has a higher average network density, limiting the transport of the components to be separated and improving the separation efficiency. Self-assembly can provide a more direct means of controlling the thickness of the PA layer, but each step of the repetitive operation needs to be controlled to the same experimental conditions to ensure layer-by-layer homogeneity.

## 4. Application

As a mainstream technology with excellent separation performance, the improvement of membrane performance will also make the membrane more widely used. Currently, the main application areas of membrane separation technology include printing and dyeing wastewater treatment, seawater desalination, removal of micropollutants, metal recovery, and resource extraction. The continuous improvement of the membrane for the application of membrane also has a very considerable prospect, but the membrane separation process also needs to be used in conjunction with other processes to achieve the desired separation effect. This section enumerates the applications and prospects of TFC-structured NF/RO in different areas and summarizes the problems and challenges in their use.

### 4.1. Applications in Different Fields

#### 4.1.1. Treatment of Industrial Wastewater

Industrial wastewater refers to wastewater, waste liquids, and cooling water generated during industrial production. It is characterized by high emissions, variety, and complexity of composition [89]. Industrial wastewater contains a wide range of substances related to raw materials, the direct discharge of which results in the loss of resources and wasted costs. Considering industrial wastewater treatment for resource recovery and reuse to reduce costs and discharge pressures is an inevitable trend for industrial wastewater. Consideration is therefore being given to increasing the number of industrial wastewater treatment processes to achieve this, with effective treatment processes not only ensuring safe discharge but also recovering water and valuable materials from the wastewater. This perspective transforms industrial wastewater from a potential environmental liability to a valuable resource, signaling a shift towards a circular approach. Effective treatment of industrial wastewater can both mitigate the environmental impacts of industrial activities and promote innovation and resilience in the face of scarce resources [90].

Membrane separation technology has great advantages in the field of industrial wastewater treatment. Because of its high separation efficiency and water recovery, and in practice, its uncomplicated operation and small footprint provide more possible treatment conditions for industrial wastewater treatment [91]. The ability of the TFC-structured NF/RO membranes to separate ions enables effective desalination and purification of industrial wastewater. However, industrial wastewater treatment is more complex, so it is necessary to combine membrane separation technology with other treatment technologies [92]. Moreover, the membranes will inevitably be contaminated to varying degrees under pressure-driven conditions, and the measures taken to address the fouling will require additional floor space and investment capital [93]. The actual treatment process also needs to take into account the specific circumstances, according to the use of water or the discharge standards of the receiving body of water, to specifically consider the use of the treatment process.

#### 4.1.2. Desalination

Desalination is the production of usable water from unfavorable water qualities such as seawater and concentrated salt water, and the main technology currently used is membrane separation technology [94]. RO membranes with a TFC structure are widely used in the desalination process and are the core process of the process prepared by desalination plants. According to the International Desalination Association (IDA), about 80% of desalination plants use RO membranes as a separation technology [95]. Compared with other traditional separation technologies, RO membranes have the highest separation factor and lowest energy consumption and have the advantage of being easily scaled up and modular, making them suitable for treating large volumes of water resources [96].

Similarly, the pressure-driven retention of most ions on one side of the membrane results in more inorganic fouling of the membranes. Considering the high salt retention rate of the NF membrane for divalent ions, the use of NF as a pre-treatment process for RO can effectively alleviate the fouling of the RO membranes and improve processing efficiency [97]. However, the fouling is only relatively transferred and does not disappear, so additional pre-treatment processes need to be considered to reduce the fouling of the membrane separation system [98]. In addition, the use of NF as a pre-treatment process requires the consideration of operating costs. NF membranes can only be operated at low pressures with high fluxes in order to minimize energy consumption [99]. Therefore, the decision to use NF membranes as a pre-treatment process should be made based on a thorough evaluation of cost and treatment effectiveness [100,101]. Desalination generally places high demands on the operating conditions in the membrane process. The operating pressure of seawater desalination is generally 5.5 MPa, which requires membranes with excellent separation properties and high mechanical strength and pressure resistance. Designed to enhance membrane separation performance while also considering pressure resistance [102]. However, higher operating pressures increase operating costs, so exploring low operating pressures is a future trend in desalination.

#### 4.1.3. Micropollutant

Micropollutants are substances that are present in small amounts but are toxic, harmful, and difficult to degrade and can be a burden on health, the environment, and the economy [103]. Nano-plastics are a type of micropollutant that poses many challenges for their control due to difficulties in quantification and degradation [104]. In recent years, it has received attention and research from ecologists, chemists, and other scientific professionals. Nano-plastics in water bodies originate from the decomposition of small plastics, and wastewater treatment plants are considered to be one of the important sources of nano-plastics. The main nano-plastics used in water treatment plants are PE, PP, and PET [105]. As an important center for the transfer of nano-plastics between humans and the natural environment and an important link in the control of the nano-plastics cycle, the removal of nano-plastics at the level of water treatment plants is of particular importance. Nano plastics in water bodies have a high specific surface area and can easily carry toxic and hazardous substances. Therefore, the removal of nano-plastics is important for human health and environmental protection.

Although most of the nano-plastics can be removed during the preliminary water treatment process, the small amount of micro-plastics and nano-plastics remaining in the effluent can still exacerbate the level of pollution in the receiving water. Therefore, further treatments for the removal of micro-plastics and nano-plastics are needed to reduce the chances of micro- and nano-plastics flowing into the natural environment. Typically, membranes are used as advanced treatment units in a treatment process flow to treat multivalent ions and some organic contaminants. The application of membrane separation technology has high efficiency for the removal of micro-plastics and nano-plastics [106]. The role played by TFC-structured membranes in the removal of micro-plastics and nano-plastics is mainly due to the small pores of the membranes that can effectively intercept micro-plastics and nano-plastics. Adsorption of micro- and nano-plastics by membranes and electrostatic repulsion can also intercept micro-plastics and nano-plastics. However, the removed micro-plastics and nano-plastics may cause secondary fouling of the membrane and increase the processing burden of the membrane. Being of similar size to the membrane pores, micro-plastics and nano-plastics can cause clogging of the membrane pores. Nano-plastics gradually generate a cake layer on the membrane surface, reducing the membrane’s separation performance and thus increasing treatment costs, which can be effectively avoided by targeted anti-fouling initiatives [107]. However, membrane separation technology as an advanced treatment unit is not designed to go out to micro-plastics and nano-plastics, resulting in the complexity of micro-plastics and nano-plastics removal not being well considered. In the future, membrane separation technology should be considered as a separate application for the removal of micro-plastics and nano-plastics on a viable economic basis. 

#### 4.1.4. Resource Recovery

The recycling of resources provides more application opportunities for the application of RO/NF membranes based on TFC structures, especially for the recycling of metals. Heavy metal pollution is the pollution of the environment by heavy metals and their compounds. Heavy metals in water bodies are mainly nickel, zinc, copper, and cadmium produced in industrial production, which are toxic, persistent, non-degradable, and bioaccumulative hazards [108]. The removal of heavy metals from water bodies is important for the protection of ecosystems and human health. Membrane separation technology for the separation of heavy metals is a simpler and more efficient recovery process than electrochemical, precipitation, adsorption, coagulation, and other technologies. The biggest advantage is that membrane separation does not produce secondary pollution [109]. The pore size of TFC-structured NF/RO membranes is suitable for separating heavy metal ions and other contaminants, but the negative charge carried on the surface of the membrane reduces the removal efficiency of the membrane for heavy metal ions. Therefore, modifying the surface of NF/RO membranes to make the membrane surface positively charged can improve the removal efficiency of heavy metal cations. The low operating pressure of NF membranes is more promising for the removal of heavy metal ions than the high operating pressure required for RO membranes.

Although TFC-structured NF membranes have achieved great success in the field of heavy metal ion removal, the actual wastewater treated contains mixed ions, increasing the treatment difficulty. Therefore, efforts should be made to develop membranes that have a significant separation effect on mixed ions to improve the separation efficiency and thus reduce the waste of resources [110]. The stability of the NF membranes for the separation process should also be considered. Accumulation of heavy metal ions on the membrane surface causes flux reduction, and the performance of the membranes decreases dramatically over a few days, so there is a need to improve the stability of the NF membrane during use. Future research extends the test cycle according to practical needs and focuses more on stability testing.

The demand for lithium as the most promising light metal in many fields is increasing year by year, especially driven by the rapid development of lithium electric vehicles [111]. One of the main ways to source lithium is by extraction from salt lakes. It is estimated that about 70 percent of lithium exists in salt lakes, but extraction of lithium ions from salt lake water faces various problems. The main problem is the interference of other ions with lithium extraction. In particular, the similarity of Mg^2+^ to its ionic hydration radius results in a lower separation ratio, making lithium extraction more challenging. TFC-structured membranes are used in magnesium–lithium separations due to their low cost and relatively high separation efficiency, especially for TFC-structured NF membranes [112]. According to the Dornan model, aperture sieving can have a relatively ideal separation effect for monovalent lithium ions and divalent magnesium ions, which has been considered the mainstream process for achieving efficient magnesium-lithium separation [113]. From the study of commercial membranes by Wen et al., it can be seen that although NF membranes can be effective in separating lithium and other substances, solute transport through the membranes is highly dependent on the distribution and solubility of the host species in the feed solution [114]. In the actual production process, according to the actual situation of the water to be separated specifically select the appropriate separation membrane.

Although TFC-structured membranes are relatively mature and commercially available for the above applications, there is a need to further consider from the perspective of raw material cost, production energy consumption, and generation efficiency so that the membranes in different fields of application of the future are bright and becomes the dominant process in the field. We also need to consider the environmental impact of the membrane in the use of the process, which should be as far as possible from the production of raw materials to the use of the whole process to strive to achieve the goal of green zero pollution.

### 4.2. Membranes Module

**Figure 4:**
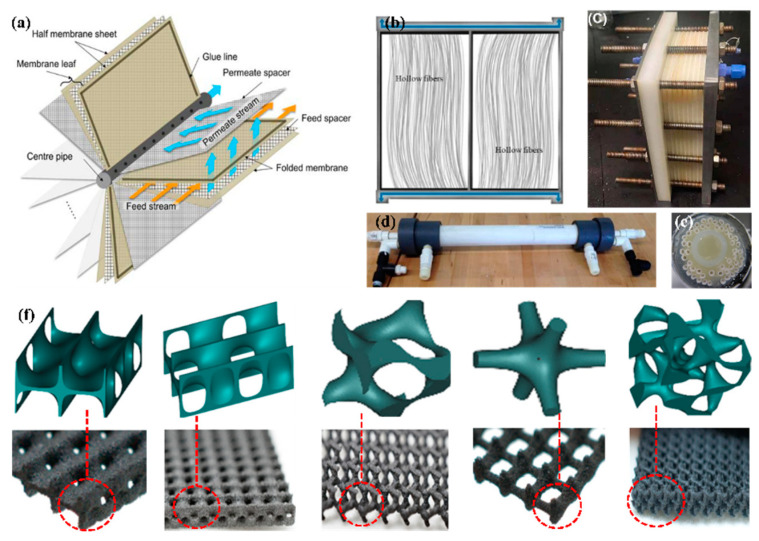
(**a**) Schematic diagram of rolled membrane module [117]; (**b**) schematic diagram of hollow fiber curtain membrane module [118]; (**c**) schematic diagram of flat membrane module [119]; (**d**,**e**) schematic diagram of hollow fiber-shell membrane module [120,121]; (**f**) 3D Printing Spacer Types [122].

In practice, the membrane sheet or filament must be mounted in a fixed frame to provide support for the membrane. Typically, the membranes and the corresponding equipment are collectively referred to as membrane modules. Membrane modules, in addition to the practical convenience of use, can also be easily replaced when they can not be used, which is conducive to cost reduction. However, membrane modules must meet certain requirements in terms of production cost, control of concentration polarization, packing density, and energy consumption [115,116]. Four types of membrane modules are commonly used: flat membrane modules (plate and frame), membrane modules in rolls (spiral wound), hollow fiber curtains, and fiber-shell modules. Schematic diagrams are shown in Figure 4a–e.

The design of membrane modules should pursue the goals of low energy consumption, high efficiency, and low cost, in addition to meeting the need to adapt to different application environments and water quality requirements. This requires the continuous improvement of the performance and long-term stability of the membranes, as well as the design of the equipment to be used to achieve optimum conditions of using [123]. The spacer placed in the module is an important part of the membrane modules that separates the membrane sheets and is placed between the feed channel and the permeate channel to support the membranes. The design of spacers used for membrane modules can improve the conditions of use of membrane modules. Because the spacer affects the mass transfer and distribution of the feed liquid and the change of concentration polarization, the structural design of the spacer can enable the membranes to have a higher mass transfer flux. However, the design of the spacer needs to follow the principle of balance between the mutual constraints issues, controlling the same pressure drop and preventing fouling while uniformly transferring the mass [124]. As the most commonly used membrane module, grid design improvements in rolled membrane modules can play a more critical role in terms of cost and wider application.

Advanced research techniques have been applied to design membrane modules, making it possible to achieve goals that are not possible with conventional preparation methods. Additive manufacturing (3D printing) technology is a rapidly emerging technology platform for many manufacturing industries in the industry, and its application to the field of separation membranes is gradually entering the research horizon [125]. For the production of membrane module spacers, 3D printing technology not only allows for the design of spacers with exotic configurations and optimization of spacer geometry but also increases production rates. However, for the realization of industrial applications, further consideration of economic costs and effectiveness of use is required. The geometries of 3D printed designs of spacers that are currently available include herringbone, stepped, helical structures, twisted bands, and multilayer structures, as shown in Figure 4f. While there may still be some unavoidable issues with quality and resolution, it has an excellent effect on performance [126]. Dang et al. designed the structure of the spacer using advanced 3D printing techniques and prepared three micro-spacers with herringbone and gyroscopic structures [127]. Although there is a slight increase in pressure drop compared with normal spacers, there is a significant increase in filtration performance and a reduction in membrane biofouling. This can be attributed to the fact that the spacers of this configuration have a better mixing effect, which helps to improve the distribution management of the feed solution, which in turn eliminates low-flow dead zones within the membrane module and increases the shear near the surface to minimize contaminant adhesion. Sreedhar et al. designed different 3D printing feed spacers based on the complex geometry of the triple periodic minimum surface (TPMS) [128]. Novel structural improvements that minimize the pressure drop in the feed channel and reduce the generation of biofilm on the membrane surface provide a bright area of research to enhance the performance of membrane modules.

Although the application of 3D printing technology to the design of membrane module structures is very innovative, the effect of temperature on print quality must be considered while using 3D printing technology, including print head temperature, print bed temperature, and ambient temperature [129,130]. The temperature of the printhead has the most direct impact on print quality. Too high a temperature may produce air bubbles, while too low a temperature may result in the material not being able to melt, and nozzle clogging may occur, impairing the quality of the printed spacer. The print bed temperature and the ambient temperature are also critical to the accuracy and detail of the printed spacer [131,132]. The right temperature ensures that the printed spacer is free from deformation and distortion. Controlling the temperature properly can make 3D printing technology more mature and allow spacers with novel structures and high productivity to be obtained.

In addition to designing the structure of the spacer, physical or chemical modification of the surface can reduce biological fouling of the surface and help increase the flux of the membrane modules. However, inhomogeneity of the surface modification of the spacer can seriously affect the service life and functional stability. For example, coating the spacer surface with a metal or metal oxide or functionalizing the surface with a hydrophilic polymer using, for example, a grafted plasma treatment. Although the modification of the surface will improve the effectiveness of the spacer, it is also necessary to consider whether the weak interaction between the modified substance and the spacer will result in a stable effect for long-term use. Ronen et al. modified the membrane surface of a spacer by depositing silver nanoparticles with good antimicrobial properties onto the spacer surface by deposition [133]. The presence of silver ions was demonstrated using numerical simulation analysis to prevent bacterial attachment and continued biofilm development in the vicinity of dead zones caused by spacers and in the vicinity of adjacent membranes. However, there is a possibility that silver-resistant bacteria may develop under long-term use. Therefore, the prevention of bio-fouling cannot rely solely on the modification of spacers, and a comprehensive approach still needs to be considered to completely solve the problem of bio-fouling. The use of metal oxides avoids the possibility of creating resistant bacteria. Thamaraiselvan et al. planted ZnO seeds on spacers by chemical bath deposition and generated ZnO nanorods by continued growth on the seed layer [134]. The nanorods grown on the spacer reduced the flux attenuation of the membrane module to less than 40%, which is good proof that the metal oxide-modified spacer can improve the resistance to biofouling. Although the modification of the spacer with metals and their oxides provides good resistance to biological fouling, it also increases the corresponding economic costs. Also, the added coating exists to cause additional fouling to the membrane module, increasing the stress on the membrane module treatment.

Despite the advances and innovations in spacer design, there is still a large discrepancy between theoretical design and practical application. In particular, the resolution of 3D printing technology seriously affects the actual performance of the product. Water quality varies during actual use, and small fluctuations may make it impossible to design a product that can fully cope with such variations. It is, therefore, not only the design of the components used in the membrane modules that are important but also the level of innovation that is needed to close the technology gap. Careful consideration should also be given to the combination with other processes to minimize the fouling of the spacer by changes in water quality to achieve long-term stability of the membrane module. 

### 4.3. Membrane Fouling and Damage

#### 4.3.1. Membrane Fouling

Membrane fouling and damage are unavoidable during the use of membranes and occur immediately after use. With the seasons and different water qualities, the type and number of substances to which the membrane is contaminated varies considerably. Membrane fouling is usually caused by organic fouling, inorganic fouling, microbiological fouling, and other types of fouling, but several types of fouling are present at the same time caused by the fouling [135]. Control of membrane fouling needs to be based on a thorough understanding of membrane fouling mechanisms. For NF and RO membranes with TFC structure, the “ridge and valley” structure on the membrane surface provides space for fouling to stay. Moreover, the charged nature of the membrane surface makes it easy to adsorb salt ions of opposite charge, which can easily cause fouling on the membrane surface and aggravate the process of membrane fouling. In addition, some organisms will stay on the surface of the membranes and cause corresponding biological fouling. Compared with other types of fouling, biological fouling is also susceptible to water quality conditions.

Some difficult-to-deal-with fouling continues to accumulate on the membrane surface and will lead to the end of the life of the membranes. Control of membrane fouling needs to take targeted countermeasures. Chemical cleaning with acidic and alkaline solutions is commonly used to reduce membrane fouling. Alkaline solvents dissolve organic matter, and acidic solutions remove scale layers [136]. However, extremely acidic and alkaline conditions can destabilize the membranes, so pH stability is also an important indicator for examining the membranes. While acid and alkaline cleaning will be effective in slowing down membrane fouling, it will not fully restore the membranes to their initial performance. The PA layer of most TFC-structured NF/RO membranes is subjected to varying degrees of chemical attack after cleaning with acid or alkaline solutions, causing membrane damage and resulting in increased flux but reduced retention. The chemical cleaning agents used are also a source of environmental pollution. Green and efficient cleaning methods are important for solving membrane fouling and environmental protection [137].

Acid and alkali solutions and some toxic and hazardous substances on the membranes cleaning to reduce membrane fouling will not only cause harm to the environment but will also increase operating costs. The development of membranes with self-cleaning functions is expected to solve this problem. Examples include photocatalytic self-cleaning, enzymatic cleaning, and osmotic backwashing [138]. Photocatalytic cleaning has been widely researched due to its green and efficient advantages, and photocatalytic materials are easy to obtain and abundant. Advanced nano-photocatalytic materials can not only enhance the permeation performance of membranes but can also be used to improve the fouling resistance of membranes. Often, it can also be combined with materials that enhance membrane properties. Quantum dots are a class of nanomaterials that can precisely modulate their spectral properties. Applying it to enhance the membrane’s resistance to fouling can give the membranes excellent self-cleaning capabilities. For example, Mi et al. prepared nanoparticles by combining carbon quantum dots with titanium dioxide and used them as an intermediate layer. The hydrophilicity of the titanium dioxide intermediate layer not only greatly improved the permeation performance of the membranes. The visible light photocatalytic ability also enables the membranes to exhibit stable and effective self-cleaning properties. Higher flux recovery and no loss of dye retention compared with conventional physical aqueous cleaning [139].

The development of membranes with self-cleaning behavior should be considered from the point of view of fully exploiting the inherent properties of the materials used. A metal–carbon matrix material is a composite material in which metal ions are introduced into the surface or structure of a carbon-based material. The addition of metals can change the original chemical, physical, and electronic properties of carbon-based materials, thus giving them new functions and applications [140]. Carbon-based materials have not only been shown to exhibit good metallic and electronic properties during application but also to enhance the properties of the substance being modified. The tantalizing prospect of introducing metal-carbon-based materials into TFC-structured membranes for self-cleaning membrane applications is aggravating new enthusiasm amongst scientists to research new materials. However, its application to the self-cleaning behavior of membranes has also become an urgent scientific innovation problem. Niu et al. used two-dimensional carbon-based materials combined with a zeolite porous structure applied to the self-cleaning function of RO membranes for desalination. It was demonstrated using computational means, such as molecular dynamics simulations, that the carbon-based material displays metallicity that facilitates the escape of already adsorbed salt ions, thus giving the membrane a self-cleaning function [141]. The use of carbon-based metal materials not only reduces the risk of environmental pollution but also provides a good foundation for the popularization of man-made materials. However, more in-depth and more practical research is needed before full-scale applications can be realized.

In addition to being used in combination with carbon-based materials to enhance the self-cleaning properties of membranes, the porous materials themselves can be used for the self-cleaning function of the membranes. MOFs, as a new class of porous solid nanoparticles, have advantages such as structural tunability and multifunctional catalytic activity. Metal-based MOF materials with the ability to be excited by visible light have been widely used in environmental remediation. Its application to the development of membrane self-cleaning function is also highly superior. Typical Fe-based MOF materials can promote the compounding of holes and electrons in the photocatalytic reaction by using reversible redox between divalent and trivalent means, thus serving the purpose of catalytic degradation. Li et al. applied MIL-53(Fe) to the preparation of TFC-structured NF membranes. The added nanoparticles not only act as nanofillers but also as photocatalysts, greatly enhancing the self-cleaning ability and permeability of the membranes [142]. The filler nature and photocatalytic ability of MOF nanomaterials have been utilized to make the application of MOF in TFC-structured membranes more widespread. Xu et al. applied Zn-TCPP-based composites to sparsely packed nanofiltration membranes with a braided shape. The nanomaterials embedded in the polyamide layer can not only significantly enhance the permeation performance of the membrane but also completely degrade the dye under the irradiation of visible light, making the membrane highly self-cleaning [143]. Research into the above self-cleaning methods needs to be accelerated to speed up their commercialization and industrial use. 

Fouling of membrane modules has a serious impact on the operation of the membrane process. The cleaning method usually used also employs alternating strong acids and strong bases. In addition to the use of the above cleaning methods, in the actual use of the application process, pre-treatment for the prevention of membrane fouling has a good effect. Relatively high-quality water conditions can extend the life of the membrane and reduce the impact of impurities on the membrane surface. A proper pre-treatment program can improve operational efficiency, reduce costs, effectively reduce membrane fouling, and improve membrane life. Examples include microfiltration, ultrafiltration, and corresponding chemical treatments. Although the treatment process is increased, it can effectively reduce the fouling caused by the membrane, increase the service life of the membrane, and reduce the replacement cost of the membranes, thus reducing the total cost of the water treatment plant in general. Pre-treatment solutions are not only important for mitigating membrane fouling but also for reducing the burden on the entire treatment process. 

#### 4.3.2. Membranes Damage

To mitigate biological fouling of membranes and to disinfect water bodies, chlorine-containing biocides are often used in response. However, these biocides are mostly oxidizing substances, which can damage the PA layer on the membrane surface, leading to a reduction in membrane flux or even a complete loss of membrane performance [144]. The most common disinfectant substances in wastewater are chlorine and its oxides. Chlorine has a strong destructive effect on the polyamide PA layer. An in-depth study of the chlorine degradation mechanism of polyamide (PA) RO membranes and the establishment of the corresponding chlorine resistance mechanism is another hot spot in the field of membrane research. TFC membranes are attacked by oxidizing agents such as chlorine and chloramines to varying degrees, causing damage. The main cause is due to the reaction between chlorine and the amide nitrogen and aromatic ring. The substances that cause the membranes to be attacked by chlorination are Cl_2_, HOCl and OCl^−^ [145]. Free chlorine attacks the H on the amide N, and then a rearrangement reaction occurs that causes the chlorine atom to transfer to the amide N, completing the substitution of the H atoms on the amide N. However, the N-Cl product is considered to be unstable and may undergo an Orton rearrangement reaction to produce cyclic chlorination products, which may damage the membrane [146]. Based on the above possible chlorination mechanisms, the strategy to enhance the chlorine resistance of membranes can mainly be carried out in the following aspects: reducing the chlorine-sensitive sites on the membrane surface, incorporating anti-chlorine compounds, and inhibiting the generation of free radicals. Removal of amine or chloride groups on the surface for the reaction reduces the activity of the PA layer to the chlorination reaction. To increase the stability and hydrophilicity of the PA layer, a hydrophilic polymer can be coated on the PA layer to promote intermolecular hydrogen bonding of the amide bonds and hinder the amide bonds from undergoing chlorination and hydrolysis reactions [147,148]. The introduction of sacrificial functional groups takes advantage of the stronger reaction between the introduced functional group and the chlorine oxidant to protect the amide bond from attack [149]. The choice of the above methods should be explored for a clear mechanism of chlorine resistance to make the methods more generalizable, and the cost of use should also be considered.

Adding a barrier layer on the surface of the PA layer can reduce the direct contact between free chlorine and the PA layer, but the choice needs to be made to select a suitable barrier layer to avoid a reduction in membrane flux. This can be achieved by modifying the PA layer with a commercial epoxy resin. However, the hydrophobicity of epoxy resin itself will reduce the membrane flux. Therefore, it is necessary to develop a barrier layer that can play a role in isolation without damaging the membrane’s performance, which is an important step in improving the chlorine resistance of the membranes [150]. The use of isolation layers does not solve this type of problem very perfectly, despite increasing the number of processing steps. Therefore, it is necessary to consider from the point of view of the reaction monomer, which is designed to resist chlorine damage from the initial reaction monomer [151]. For example, Peng et al. replaced the modified PIP monomer with conventional PIP. This allowed the primary amine (-NH_2_-) originally contained in the diamine monomer to react with the chlorine chloride monomer using Secondary Amine, reducing the chlorine-sensitive amide bond in the membranes, which can effectively avoid the attack of free chlorine [152]. The development of new monomers for chlorine resistance advances the innovation of new materials, and it is also possible to consider both the improvement of performance and the enhancement of chlorine resistance as the innovation point of new materials.

Extending the life of the membranes is an important study in making the best use of the membranes, and damage or fouling of the membranes is, to some extent, irreparable. It is very difficult to restore membranes to their original state using cleaning, which results in an average membrane module life of about six years [153]. Currently, based on the dependence on desalinated water, the number of membrane modules in use is relatively large, and the annual replacement rate is as high as 20%, resulting in a large amount of wastage of membrane modules [154]. The typical disposal of discarded membrane modules is to landfill them as solid waste. As a solid waste, the first consideration should be resource recovery, so consider simplifying the means of disposal by reusing the membranes or membrane modules for other purposes or recycling discarded membrane modules. Resource recovery processing allows for the recycling of membrane-related materials to practice sustainable development strategies [155].

Fouling and structural damage to the PA layer leads to the eventual loss of membrane performance. If the contaminated PA layer is separated from the support layer, the reuse of resources can be achieved by converting the TFC-structured NF or RO membranes into MF or UF. Contreras-Martínez et al. treated the PA layer on the membrane surface with NaClO, and PSF on PE substrates was recovered using different solubilities and different exposure times to dissolve the PA layer obtained. The recovered PSF membranes were then re-interfacially polymerized to prepare FO membranes that could be used in conjunction with other processes [156]. Recycled membranes have been shown to have higher fluxes compared with commercial membranes. The reuse of waste films not only achieves recycling and sustainability but also minimizes economic losses and reduces ecological impacts.

## 5. Conclusions

**Figure 5:**
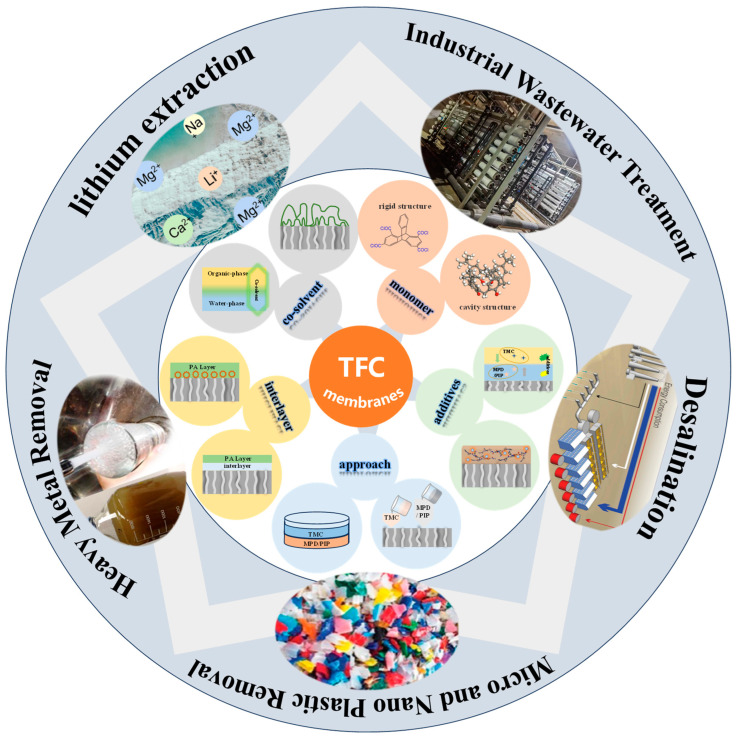
Summary diagram of modification and application (Actual application pictures can be referred to [112,154,157,158]).

This paper introduces the basic principles and the latest research progress of NF and RO membranes based on TFC structure and systematically summarizes the applications of TFC-structured membranes in different fields. The schematic diagram of the summary is shown in Figure 5. Separation layers are prepared based on IP as a key layer in determining membrane performance. From the selection of reactive monomers to the updating of preparation methods, innovative ways have been sought to improve performance. The molecular design of the reactive monomer can lead to a high breakthrough in membrane performance. Reactive monomers with rigid structures have been designed and synthesized to improve membrane permeation performance, but there is still a need to consider the economic cost. The modification of reactive two-phase solutions has also been studied. Changing the reaction solvent, adding co-solvents, adding nanoparticles, etc., can significantly change the nature of the two-phase solution and the miscible zone and thus change the IP reaction process. To accurately design the membrane structure and further improve the separation performance of the membranes, the membrane preparation method is constantly being innovated to make up for the shortcomings of the traditional preparation method. However, in general, the above modifications revolve around the basic principle of IP. Therefore, a clearer understanding of the membrane synthesis mechanism and further research are needed. Novel synthetic monomers and preparation processes will also be developed as never before. TFC-structured NF/RO membranes have been used in desalination, resource recovery, heavy metal extraction, industrial wastewater treatment, and micropollutant treatment. However, as the technology for preparing membranes continues to improve, the practical applications will continue to evolve.

## 6. Outlook

Although the design of membrane structures, the development of new membrane materials, and the ease of manufacturing will continue to expand the areas of application for membranes, there is a need for a better understanding of membrane structure-property relationships and the development of protocols to precisely regulate membrane structure. From the membranes module, membranes process, membranes system, and other continuous optimization, to give full play to the role of the membranes in low pollution, high performance, low energy consumption process, to achieve the application of the maximum membrane. Moreover, the current enhancement of membrane performance is basically at the laboratory level, and subsequent efforts should be devoted to the expanded production of membranes as well as the development of process use and long-term stability. Particular attention has been given to the application of advanced research techniques to the field of membranes. Although 3D printing technology, kinetic monitoring, and other digital technologies have been involved, a better fit with them is still an obstacle to advancing membrane technology. Various numerical methods such as computational fluid dynamics (CFDs), mathematical approximation, and mechanical modeling have been widely used and well-evaluated in membrane preparation processes. A better understanding of membrane separation processes can be gained by simulating and predicting trends in mass transfer and chemical reactions between chemicals and membranes. In addition to modeling the membrane separation process, the application of model predictions to membrane fouling can reveal specific fouling mechanisms and enable the prevention of membrane fouling. Better integration of advanced technologies with membrane preparation also requires continuous exploration by a wide range of researchers and actively promotes the development of mutual integration of various disciplines.

## Figures and Tables

**Table 1:** Summary of amine monomers and acyl chloride monomers.

Type	Name	Framework	Operating Condition	Performances	Ref.
amine monomer	m-Phenylenediamine (MPD)	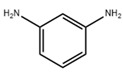	1.5 MPa, 25 °C 2000 ppm NaCl	45–60 L/m^2^h 98.8%	[37]
	piperazine (PIP)	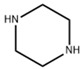	3.5 bar, 500 mg/L MgSO_4_	14.3 (L/m^2^hbar) (98.6%)	[38]
	Tris(2-aminoethyl)amine (TAEA)	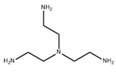	1.0 MPa, 25 ℃ 2000 ppm	135.9 (L/m^2^h) S_Li_^+^/_Mg_^2+^ = 25.94	[39]
	1,3,5(Tri-piperazine)-triazine (TPT)	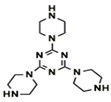	100 psi, 25 ± 1 °C 2000 ppm MgSO_4_	8.68 (L/m^2^hbar) 98.6%	[40]
	m-phenylenediamine-5-sulfonic acid (SMPD)	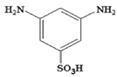	15 bar, 2000 ppm, NaCl	30.0–55.7 (L/m^2^hbar) 47–94%	[41]
	1,3cyclohexanebis(methylamine) (CHMA)	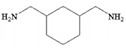	10 bar, 2000 ppm, NaCl	56 (L/m^2^hbar) 77%	[42]
Chloride monomer	Trimesoyl chloride (TMC)	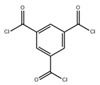	1.6 MPa, 25 °C 2000-ppm NaCl	3.31 ± 0.10(L/m^2^hbar) 99.3 ± 0.1%	[34]
	terephthaloyl chloride (TPC)	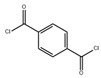	10 bar, 25 °C	7.64 ± 0.1 (L/m^2^hbar)	[43]
	5-isocyanato-isophthaloyl chloride (ICIC)	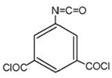	1.55 MPa, 25 °C NaCl	----	[44]
	5-chloroformyloxy-isophthaloyl chloride (CFIC)	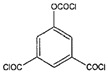	1–3 MPa 25 °C 500–8000 mg/L NaCl	20 (L/m^2^h) 50.2%	[45]
	3,4′,5-biphenyl triacyl chloride (BTRC)	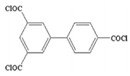	20 bar, 2000 ppm, NaCl	33 (L/m^2^h) 98.9%	[46]
	3,3′,5,5′-biphenyltetraacyl chloride (BTEC)	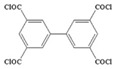	55 bar, 32,800 ppm, NaCl	30.2–48.3 (L/m^2^h) 99.3–99.7%	[47]
